# Supplementary material for: Mechanism of mammalian transcriptional repression by noncoding RNA
Source: Nat Struct Mol Biol. 2025 Jan 6;32(4):607–12. doi: 10.1038/s41594-024-01448-7 (PMC11996674; doi:10.1038/s41594-024-01448-7)
Supplement: Supplementary file 1 — Reporting Summary [file 41594_2024_1448_MOESM1_ESM.pdf]

nature portfolio

Corresponding author(s): Carrie Bernecky

Last updated by author(s): Sep 19, 2024

Reporting Summary

Nature Portfolio wishes to improve the reproducibility of the work that we publish. This form provides structure for consistency and transparency in reporting. For further information on Nature Portfolio policies, see our Editorial Policies and the Editorial Policy Checklist.

Statistics

For all statistical analyses, confirm that the following items are present in the figure legend, table legend, main text, or Methods section.

n/a	Confirmed	
☐	☒	The exact sample size (*n*) for each experimental group/condition, given as a discrete number and unit of measurement
☐	☒	A statement on whether measurements were taken from distinct samples or whether the same sample was measured repeatedly
☐	☒	The statistical test(s) used AND whether they are one- or two-sided<br>*Only common tests should be described solely by name; describe more complex techniques in the Methods section.*
☒	☐	A description of all covariates tested
☒	☐	A description of any assumptions or corrections, such as tests of normality and adjustment for multiple comparisons
☐	☒	A full description of the statistical parameters including central tendency (e.g. means) or other basic estimates (e.g. regression coefficient) AND variation (e.g. standard deviation) or associated estimates of uncertainty (e.g. confidence intervals)
☐	☒	For null hypothesis testing, the test statistic (e.g. F, t, r) with confidence intervals, effect sizes, degrees of freedom and P value noted<br>*Give P values as exact values whenever suitable.*
☒	☐	For Bayesian analysis, information on the choice of priors and Markov chain Monte Carlo settings
☒	☐	For hierarchical and complex designs, identification of the appropriate level for tests and full reporting of outcomes
☒	☐	Estimates of effect sizes (e.g. Cohen's *d*, Pearson's *r*), indicating how they were calculated

Our web collection on statistics for biologists contains articles on many of the points above.

Software and code

Policy information about availability of computer code

Data collection	SerialEM 3.8, SerialEM 3.9, EPU 2.11
Data analysis	Warp 1.0.9, RELION 3.1, CTFFIND4, Topaz 0.2.5, CryoSPARC 3.2, Coot 0.9.6, UCSF Chimera 1.16, UCSF ChimeraX 1.6, Isolde 1.6, Phenix 1.20.1, DeepEMhancer 20210511, GraphPad Prism 9, ImageQuant TL 10.2, ImageJ (version 154.g), DiscoverMP 2.3

For manuscripts utilizing custom algorithms or software that are central to the research but not yet described in published literature, software must be made available to editors and reviewers. We strongly encourage code deposition in a community repository (e.g. GitHub). See the Nature Portfolio guidelines for submitting code & software for further information.

Data

Policy information about availability of data

All manuscripts must include a data availability statement. This statement should provide the following information, where applicable:

- Accession codes, unique identifiers, or web links for publicly available datasets
- A description of any restrictions on data availability
- For clinical datasets or third party data, please ensure that the statement adheres to our policy

Cryo-EM maps for Pol II-Alu-LA, Pol II-Alu-RA, Pol II-minimal-Alu-RA canonical conformation, and Pol II-minimal-Alu-RA alternative conformation were deposited to the EM Data Bank under the accession codes EMD-18367, EMD-18375, EMD- 18371, and EMD-18376. Model coordinates for the Pol II-AluRA polymerase core and

Pol II-minimal Alu-RA complex were deposited to the PDBe under the accession codes 8QEP and 8QEQ. Previously published and publicly accessible model coordinates are available from the PDBe under the accession codes 5OIK and 5IYB.

Research involving human participants, their data, or biological material

Policy information about studies with human participants or human data. See also policy information about sex, gender (identity/presentation), and sexual orientation and race, ethnicity and racism.

Reporting on sex and gender	n/a
Reporting on race, ethnicity, or other socially relevant groupings	n/a
Population characteristics	n/a
Recruitment	n/a
Ethics oversight	n/a

Note that full information on the approval of the study protocol must also be provided in the manuscript.

Field-specific reporting

Please select the one below that is the best fit for your research. If you are not sure, read the appropriate sections before making your selection.

☒ Life sciences ☐ Behavioural & social sciences ☐ Ecological, evolutionary & environmental sciences

For a reference copy of the document with all sections, see nature.com/documents/nr-reporting-summary-flat.pdf

Life sciences study design

All studies must disclose on these points even when the disclosure is negative.

Sample size	No sample size was calculation was performed. Biochemical assays were performed in triplicate to allow statistical analysis of the data. Number of collected micrographs was chosen based on expected heterogeneity and flexibility of the analyzed complex and the availability of microscope resources.
Data exclusions	No data were excluded.
Replication	Sample preparation and analysis were reproducible. For each sample, one small and one large data set were collected. Initial reconstructions from small data sets resembled the analysis of the full data sets.
Randomization	During 3D refinement, cryo-EM data were split randomly into two halves for gold-standard FSC determination. Biochemical experiments were not randomized, but contained appropriate controls.
Blinding	Blinding is not relevant or feasible for these types of experiments. The biochemical and structural experiments presented here are rigorously quality controlled through other criteria and mechanisms as accepted within the field.

Reporting for specific materials, systems and methods

We require information from authors about some types of materials, experimental systems and methods used in many studies. Here, indicate whether each material, system or method listed is relevant to your study. If you are not sure if a list item applies to your research, read the appropriate section before selecting a response.

Materials & experimental systems

n/a	Involved in the study	
☐	☒	Antibodies
☒	☐	Eukaryotic cell lines
☒	☐	Palaeontology and archaeology
☐	☒	Animals and other organisms
☒	☐	Clinical data
☒	☐	Dual use research of concern
☒	☐	Plants

Methods

n/a	Involved in the study	
☒	☐	ChIP-seq
☒	☐	Flow cytometry
☒	☐	MRI-based neuroimaging

Antibodies

Antibodies used	8WG16 (hybridoma line provided by N. Thompson and R. Burgess)
Validation	This antibody was used to purify RNA polymerase II.

Animals and other research organisms

Policy information about studies involving animals; ARRIVE guidelines recommended for reporting animal research, and Sex and Gender in Research

Laboratory animals	This study did not involve laboratory animals.
Wild animals	This study did not involve wild animals.
Reporting on sex	n/a
Field-collected samples	This study did not involve field samples
Ethics oversight	This project does not raise ethical issues. Pig thymus (used for protein purification) is sourced from animals approved for food consumption through an officially approved facility in Sieghartskirchen, Lower Austria.

Note that full information on the approval of the study protocol must also be provided in the manuscript.
