# Supplementary material for: Mechanism of mammalian transcriptional repression by noncoding RNA
Source: Nat Struct Mol Biol. 2025 Jan 6;32(4):607–12. doi: 10.1038/s41594-024-01448-7 (PMC11996674; doi:10.1038/s41594-024-01448-7)

c **Transcription inhibition by Alu-RNA**

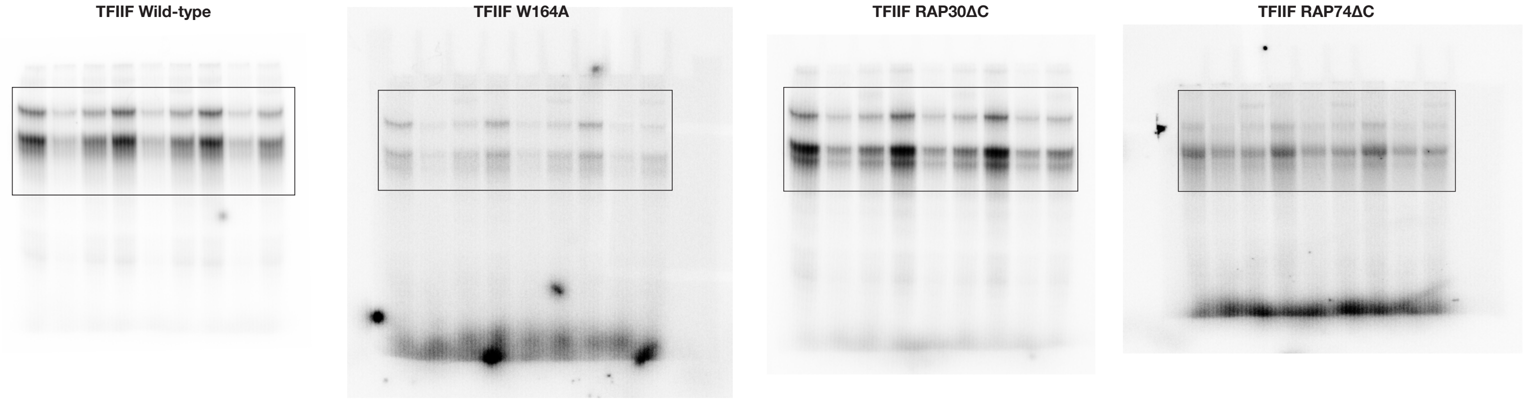

Supplement: Supplementary file 5 — Uncropped gels analyzed in Fig. 2c. Cropped versions are shown in Extended Data Fig. 6e. [file 41594_2024_1448_MOESM5_ESM.pdf]
