# Supplementary figures and images for: Mechanism of mammalian transcriptional repression by noncoding RNA
Source: Nat Struct Mol Biol. 2025 Jan 6;32(4):607–12. doi: 10.1038/s41594-024-01448-7 (PMC11996674; doi:10.1038/s41594-024-01448-7)

**a**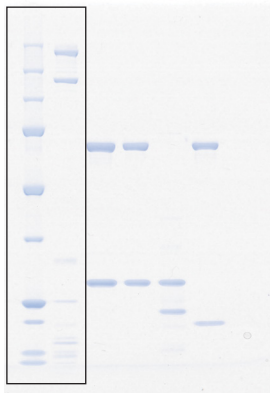**b**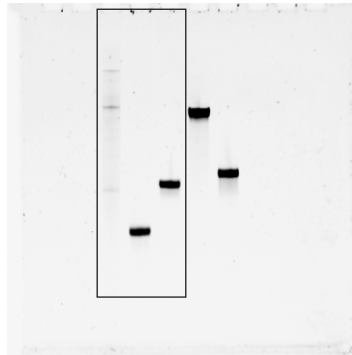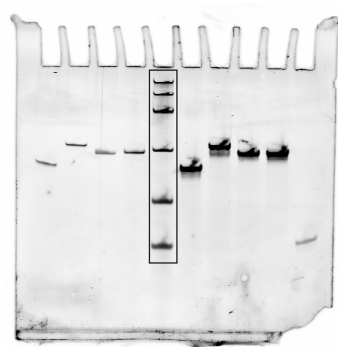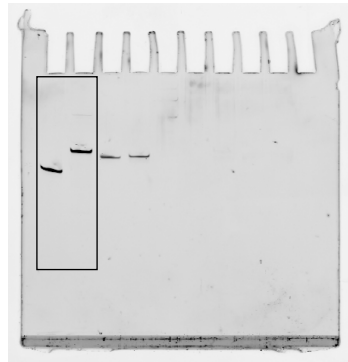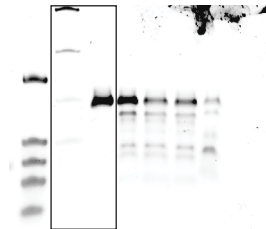

Supplement: Supplementary file 6 — Uncropped gels shown in Extended Data Fig. 1a,b. [file 41594_2024_1448_MOESM6_ESM.pdf]

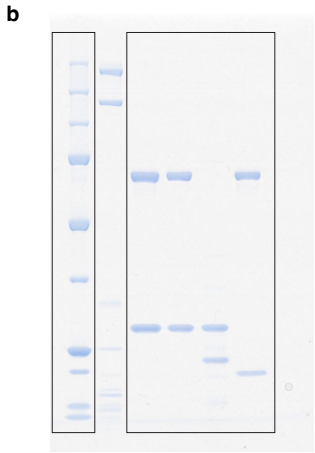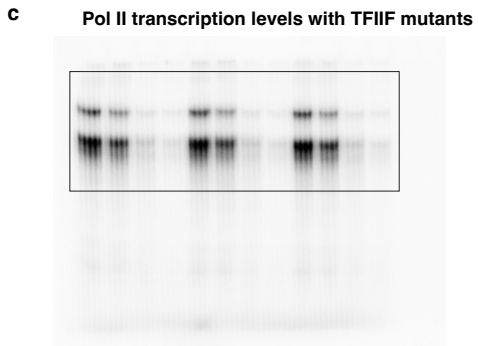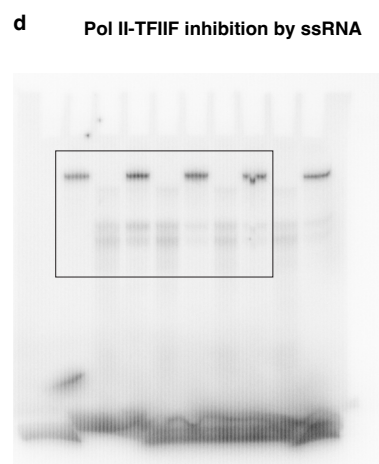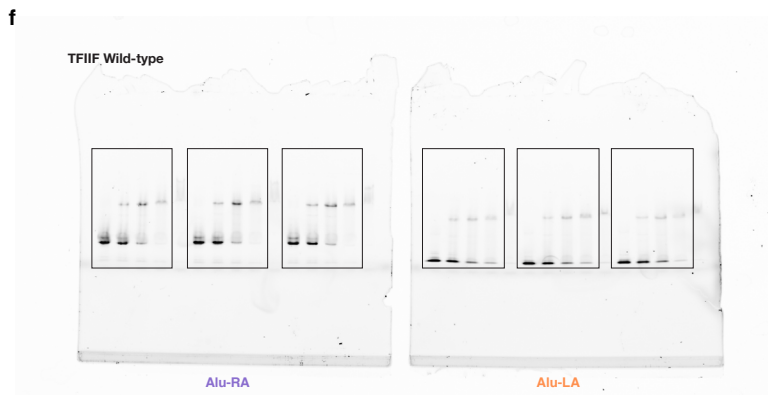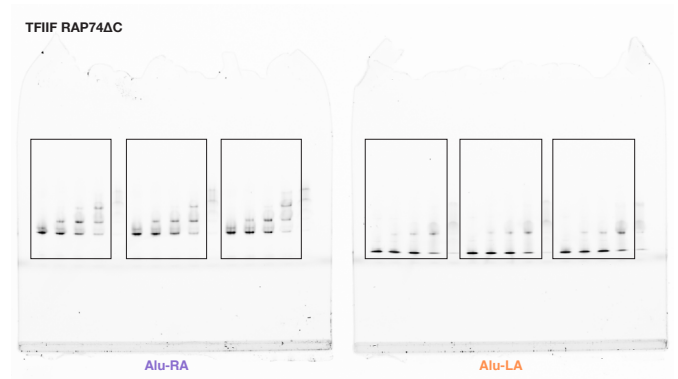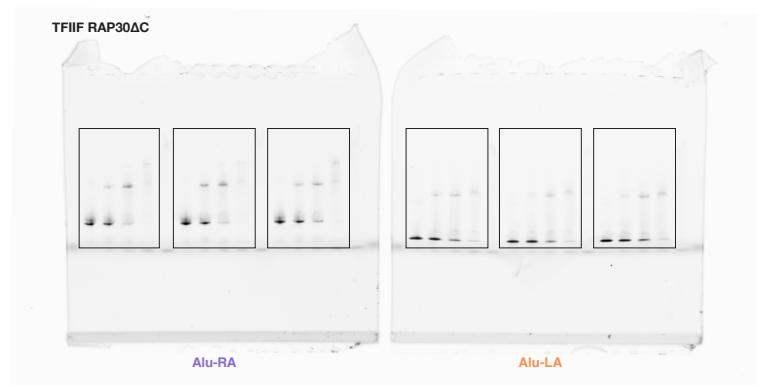

Supplement: Supplementary file 8 — Uncropped gels shown in Extended Data Fig. 6. [file 41594_2024_1448_MOESM8_ESM.pdf]
